# Effect of Dietary *Cannabis sativa* L. Residue Supplementation on Meat Quality and Flavor-Enhancing Free Amino Acids in Broiler Chickens

**DOI:** 10.3390/ani15050759

**Published:** 2025-03-06

**Authors:** Yusup Sopian, Katatikarn Sahatsanon, Apinya Satsook, Chaiwat Arjin, Korawan Sringarm, Chompunut Lumsangkul, Panneepa Sivapirunthep, Chanporn Chaosap

**Affiliations:** 1 Doctoral Program in Innovative Tropical Agriculture, School of Industrial Education and Technology, King Mongkut’s Institute of Technology Ladkrabang, Bangkok 10520, Thailand; 65036096@kmitl.ac.th (Y.S.); 65036095@kmitl.ac.th (K.S.); 2 Office of Research Administration, Chiang Mai University, Chiang Mai 50200, Thailand; apinya.satsook@cmu.ac.th; 3 Department of Animal and Aquatic Sciences, Faculty of Agriculture, Chiang Mai University, Chiang Mai 50200, Thailand; chaiwat.arjin@cmu.ac.th (C.A.); korawan.s@cmu.ac.th (K.S.); 4 Department of Animal Science, National Chung Hsing University, Taichung 40227, Taiwan; lumsangkul@nchu.edu.tw; 5 Department of Agricultural Education, School of Industrial Education and Technology, King Mongkut’s Institute of Technology Ladkrabang, Bangkok 10520, Thailand; panneepa.si@kmitl.ac.th

**Keywords:** drip loss, shear force, fat, fatty acids, aspartic acid

## Abstract

This study investigated the effects of dietary *Cannabis sativa* residues (CR) on broiler growth and meat characteristics. A total of 256 one-day-old male Ross-308 broilers were randomly assigned to four treatments: a basal diet (control) and diets containing 0.5%, 1%, and 2% CR. CR supplementation had no effect on growth performance, survival rate, or European production index but reduced average daily feed intake (*p* < 0.01). No significant differences were found in meat pH, color, drip loss, thawing loss, cooking loss, or shear force (*p* > 0.05). However, the ΔE values (0.5% CR: 3.97, 1.0% CR: 3.71, 2.0% CR: 4.95) indicated perceptible color differences compared to the control. CR significantly reduced C12:0, C20:1n9, and C22:1n9 fatty acids (*p* < 0.05) while increasing free amino acids, including aspartic acid, serine, proline, methionine, and phenylalanine (*p* < 0.05). It also increased moisture content and decreased fat content, especially at 2% CR (*p* < 0.05). In conclusion, CR supplementation improves the flavor and nutritional value of broiler meat and is a potential alternative to conventional feed additives.

## Simple Summary

The objective of this study was to investigate the effects of *Cannabis sativa* residues (CR) on broiler growth, meat quality, and nutritional composition. The results showed that while CR supplementation did not impact growth performance, it improved meat quality by reducing fat content and enhancing umami flavor through elevated free amino acid levels, thereby increasing the overall nutritional value of the meat.

## 1. Introduction

The increasing global consumption of poultry meat is driven by its high protein, low fat, and favorable fatty acid composition. By 2032, poultry is expected to account for 41% of global meat protein consumption, with consumption expected to increase by 15% [1]. Among poultry species, broiler chickens are particularly prized for their rapid growth, larger breast muscles, and nutritional benefits, including a higher polyunsaturated fatty acid (PUFA) content than beef and pork [2]. However, broiler production faces several challenges, including susceptibility to oxidative stress, which can negatively impact health, production efficiency, and meat quality [3]. These challenges require innovative strategies to improve broiler meat quality while maintaining production efficiency.

Adding bioactive compounds is a possible strategy for improving growth performance and meat quality. *Cannabis sativa* L., a plant with multiple uses and historical significance, is promising for this purpose. Traditionally, it has been used for its anti-inflammatory properties, as an appetite stimulant, and in treating immune system disorders [4]. It has been reported that *C. sativa* contains more than 150 cannabinoids and a wide range of other substances, including flavonoids, terpenoids, and alkaloids, which exhibit anti-inflammatory, antimicrobial, and neuroprotective properties [4,5]. In poultry nutrition, recent research has linked cannabis-derived cannabidiol diets to reduced spoilage volatile compounds in meat [6] and improved body weight, carcass characteristics, and meat quality in stressed chickens [7].

In 2019, Thailand became the pioneer in Southeast Asia by legalizing *C. sativa* for medical and research purposes [8]. Most of the phytochemicals of *C. sativa* are currently extracted from the plant’s flowers [5], while the leaves, stems, branches, and roots remain residues. Identifying practical applications for these residues is critical to promoting zero-waste practices in the cannabis industry. These residues often contain bioactive compounds with potential medicinal and nutritional benefits. According to Balenović et al. [9], adding *C. sativa* leaves to broiler diets may deliver promising antimicrobial and immunomodulatory effects. In a previous study, we found that *C. sativa* residues (CR) from the leaves and stalks contain high levels of bioactive compounds, such as cannabidiol, tetrahydrocannabinol, and total phenols, which may offer antimicrobial, antioxidant, and anti-inflammatory benefits [10]. In addition, the supplementation of broiler diets with CR improved intestinal health and the oxidative stability of plasma [10], indicating its potential as a valuable feed additive. However, its effects on broiler meat quality have not been thoroughly investigated. In this study, we hypothesized that CR supplementation would enhance broiler chicken meat quality by improving its chemical composition, nutritional value, and sensory attributes. Therefore, the purpose of this experiment was to investigate the potential of CR as an alternative feed additive for broiler diets, with a particular focus on its effects on meat quality, fatty acid profile, free amino acids, and ribonucleotides.

## 2. Materials and Methods

### 2.1. Animal Ethics Statement

The study was approved by the Animal Care and Use Committee of the Faculty of Agriculture at Chiang Mai University (Protocol No. AGIACUC015/2665) and complied with the guidelines for the care and use of animals for scientific purposes established by the National Research Council of Thailand [11].

### 2.2. Animals, Treatments, and Experimental Design

The study conducted by Sopian et al. [10] involved 256 one-day-old male Ross 308 chicks, which were randomly divided into four experimental groups with eight replicates of eight chicks each. The chicks were housed in an enclosed evaporation house containing 32 pens (120 × 100 × 50 cm) with rice husk bedding. They had ad libitum access to feed and water, which was provided via feeding troughs and nipple drinkers. In the first week there was continuous light, after which a lighting regime was introduced with 16 h of light and 8 h of darkness. The lighting was provided by incandescent lamps with a yellow light source and an intensity of 10 lux. All birds were kept under constant environmental conditions throughout the study.

The experimental diets consisted of a control diet without CR powder (0% CR) and three diets with 0.5%, 1%, and 2% CR added. The basal diet was a commercial corn–soybean meal mixture formulated according to the nutritional guidelines for broilers [12]. The detailed chemical composition is shown in Table 1 [10].

### 2.3. Growth Performance

After three days of acclimatization, the birds were then fed diets containing various amounts of CR until they were 40 days old. Body weight (BW) and feed intake (FI) were recorded using a digital weighing scale (Sunford ACS-30-FC31, Sunford, Thailand) with a measurement accuracy of ±1 g. Feed intake was determined by subtracting the leftover feed from the total feed offered. During the study, growth performance parameters were evaluated as follows: (1) Initial (IBW) and final body weight (FBW) were recorded to evaluate growth. (2) For average daily feed intake (ADFI), feed intake per bird was measured daily in grams. (3) Average daily gain (ADG) was measured as weight gain per day (4) Feed conversion ratio (FCR) was measured as weight gain per unit of feed consumed [13]. The survival rate, also known as livability, is the proportion of animals that remain alive throughout the rearing period. The European Production Index (EPI) was calculated using the following formula [14]:

$$EPI=\frac{Average\;daily\;gain\;(g/d)\times \%Livability}{FCR\times 10}\times 100$$

### 2.4. Meat Quality Analysis

#### 2.4.1. Sample Collection

On day 40, two birds from each replicate (total of 64 birds, 16 per treatment) were randomly chosen for slaughter by dislocating the neck, scalded at 60 °C for 3 min, de-feathered with a drum picker, and manually eviscerated after being chilled in an ice bath for 45 min. The pectoral muscle of the right side of each carcass was examined for its physical characteristics. Meanwhile, the left side of the pectoral muscle was removed from one chicken of each replicate (total of 32 birds, 8 per treatment) to analyze fatty acids, free amino acids, chemical composition, and ribonucleotide content.

#### 2.4.2. Physical Properties

The physical properties of meat were determined as described by Chaosap et al. [15]. The pH was measured 3 and 24 h postmortem at the cranial part using a portable pH meter (SG2—ELK Seven Go™, Mettler Toledo, Shanghai, China). Before taking the measurements, the pH meter was calibrated using buffers with pH values of 4.01 and 7.01 to ensure accuracy. Meat color was assessed using a spectrophotometer (CM-5, Konica Minolta, Osaka, Japan) calibrated with a standard white plate (Y = 93.7, x = 0.3160, y = 0.3325) provided by the manufacturer. The color was measured on the meat surface at three locations using a d/8° viewing geometry, an aperture diameter of 8 mm, and a standard D65 illuminant. The CIE L, a*, and b* values were recorded under a 10° observer angle. The ΔE value (color difference) is calculated using the formulas [16]:

$$\Delta E=\sqrt{{\left(L_{2}-L_{1}\right)}^{2}+{\left(a_{2}-a_{1}\right)}^{2}+{\left(b_{2}-b_{1}\right)}^{2}\;}$$

where

L_1_, a_1_, b_1_ were the color of the control sample;
L_2_, a_2_, b_2_ were the color of the treatment sample.

Drip loss was measured by weighing the meat samples, placing them in plastic bags, hanging them at 4 °C for 24 h, and then blotting and reweighing. The thawing loss was calculated by weighing the samples before freezing at −20 °C for 7 days, thawing at 4 °C for 24 h, and reweighing. Cooking loss was determined by heating the samples in a water bath (Memmert, Buchenbach, Germany) to 70 °C for 20 min. The samples were then cooled under running tap water for approximately 30 min until they reached room temperature, after which they were reweighed. Before cooking loss measurement, the samples were stored at −20 °C for 7 days and thawed at 4 °C for 24 h before analysis. All measurements were conducted for 8 days post-slaughter. After cutting the cooked samples along the muscle fibers into 1 × 2 × 1 cm pieces, the samples were then cut parallel to the fiber orientation of the cooked sample using a Texture Analyzer (TA.XT Plus, Stable Micro Systems, Texture Technologies Corp., Surrey, UK).

#### 2.4.3. Fatty Acid Composition

Fatty acid methyl esters (eight replicates per treatment) were prepared following Morrison and Smith [17] and analyzed using a Shimadzu GC-2030 gas chromatograph (Kyoto, Japan) with flame ionization detector (GC-FID) and a capillary column (0.25 mm × 100 m × 0.25 µm, RT-2560, RESTEK, Bellefonte, PA, USA). Helium was used as the carrier gas, with the injector and detector set to 250 °C. The oven temperature was programmed from 100 °C (held for 4 min) to 240 °C at 3 °C/min and then held for 20 min. A 1 µL sample was injected, and chromatographic data were analyzed using Lab Solution software https://www.shimadzu.com/an/products/software-informatics/labsolutions-series/index.html (accessed on 4 March 2025) (Shimadzu, Kyoto, Japan). Peaks were identified based on retention times compared to standard mixtures, and results were expressed as grams of individual fatty acids per 100 g of total fatty acids [18].

#### 2.4.4. Free Amino Acids

Following AOAC [19] and ISO [20] methods, free amino acids (FAA) were analyzed by homogenizing 1 g of sample with 25 mL of 70% ethanol, centrifuging at 10,000 rpm for 20 min, and evaporating the supernatant before dissolving it in 2 mL borate buffer. After derivatization and filtration (0.45 µm), the samples were analyzed by HPLC (Shimadzu HPLC LC-20A, Japan) equipped with an Ultra C18 column (5 µm, 250 × 4.6 mm) and fluorescence detection set at 263 nm (excitation) and 313 nm (emission). FAA content was expressed in g per 100 g of sample. For free amino acids that are related to human taste classification, sweet = glycine + alanine + proline, serine + threonine, bitter = histidine + arginine + isoleucine + leucine + lysine + phenylalanine + valine, and umami = glutamic acid + aspartic acid [21].

#### 2.4.5. Chemical Composition and Ribonucleotide Content

One bird per replicate (eight birds per treatment) was selected to determine breast meat moisture, total ash, crude protein, and ether extract content according to AOAC methods [19]. Chaosap et al. [15] described the method to determine the ribonucleotide content. In brief, 6 mL of chilled 0.6 M perchloric acid was added to 1 g of breast muscle and homogenized at 10,000 rpm. The homogenate was neutralized with 0.8 M KOH and KH_2_PO_4_ buffer to a pH of 7–8, diluted with HPLC water to a final volume of 25 mL, and centrifuged at 10,000 rpm at 4 °C for 10 min (Hitachi CR 22N centrifuge, Shinagawa, Japan). The supernatant was analyzed for hypoxanthine, inosine, inosine monophosphate (IMP), and guanosine monophosphate (GMP) using a Chromaster HPLC system (Hitachi, Tokyo, Japan) with a UV detector at 210 nm. A TSK Gel Amide-80 column (Tosoh, Tokyo, Japan) and a mobile phase in a ratio of 70:30 acetonitrile to KH_2_PO_4_ buffer facilitated the separation. Ribonucleotide concentrations were calculated using a standard curve generated from external standards.

### 2.5. Statistical Analysis

A completely randomized design was used in this study, with 8 replicates of 8 chicks per treatment group, including 4 CR supplementation levels. The slaughtered chickens were considered as experimental units per treatment for physical properties (*n* = 16), fatty acids (*n* = 8), free amino acids (*n* = 8), chemical composition (*n* = 8), and ribonucleotide content (*n* = 6). The statistical model used was Yij = µ + Ti + eij, where Yij was the dependent variable, µ was the overall mean, Ti was the CR supplementation levels (0%, 0.5%, 1%, and 2%), and eij was a random error. Data were analyzed with SPSS v.29.0 (SPSS Inc., Chicago, IL, USA) using one-way ANOVA, and Tukey’s post hoc test identified significant differences (*p* < 0.05) between treatments.

## 3. Results

### 3.1. Growth Performance

**Figure 1:**
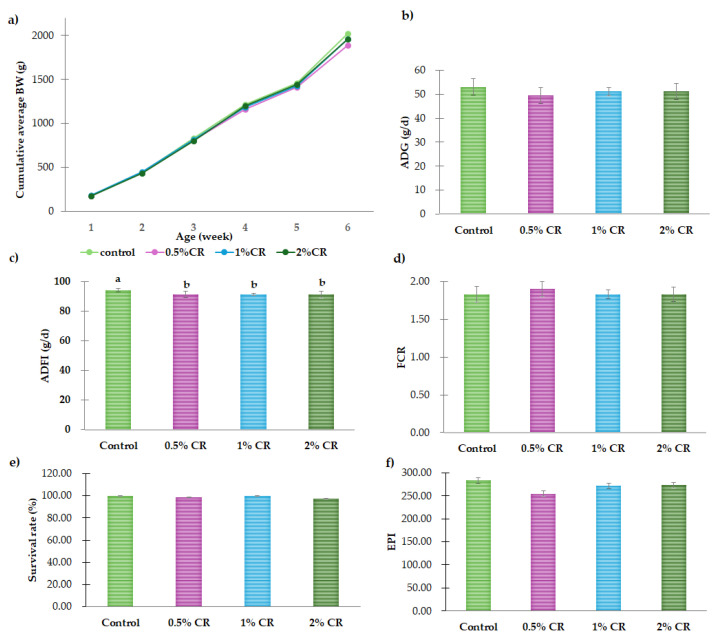
The growth performance, survival rate, and European Production Index (EPI) of broilers fed diets containing different levels of *C. sativa* residues during the experimental period: (**a**) cumulative average body weight (weeks 1–6), (**b**) average daily gain (ADG), (**c**) average daily feed intake (ADFI), (**d**) feed conversion ratio (FCR), (**e**) survival rate, and (**f**) EPI index. Graph bars with different superscript letters (a, b) indicate significant differences among the experimental treatments (*p* < 0.01).

The cumulative average body weight of broilers from day 1 to day 40 did not differ significantly between birds fed different levels of CR (Figure 1). CR supplementation had no effect on ADG (*p* > 0.05) but significantly reduced average daily feed intake (ADFI) compared to the control group (*p* < 0.01). Additionally, there were no significant changes in survival rate or the EPI index (*p* > 0.05).

### 3.2. Physical Properties

The physical properties of breast meat, including pH, color, drip loss, thawing loss, cooking loss, and shear force, were not affected by CR supplementation (*p* > 0.05), as indicated in Table 2. The ΔE calculation provides a clear indication of whether the observed color differences are perceptible to consumers. The ΔE values for Treatments 2 (3.97), 3 (3.71), and 4 (4.95) suggest that the color differences, compared to the control group, are noticeable to the human eye.

### 3.3. Fatty Acid Composition

CR supplementation significantly reduced the concentrations of lauric acid (C12:0), gondoic acid (C20:1n9), and erucic acid (C22:1n9) (*p* < 0.05) in breast meat (Table 3). However, dietary *C. sativa* residue did not affect the total concentrations of saturated fatty acids (SFA), monounsaturated fatty acids (MUFA), PUFA, omega-6, or omega-3 (*p* > 0.05).

### 3.4. Free Amino Acids

CR supplementation significantly increased aspartic acid (Asp), serine (Ser), proline (Pro), methionine (Met), phenylalanine (Phe), and total non-essential amino acid (NEAA) content (*p* < 0.05). In contrast, glycine (Gly) content was significantly decreased (*p* < 0.05), as shown in Table 4. The total content of essential amino acids (EAA) was not affected by dietary CR. CR supplementation significantly increased aspartic acid (Asp) (*p* < 0.05) and also tended to increase glutamic acid (*p* = 0.08), both of which are associated with umami taste.

### 3.5. Chemical Composition and Ribonucleotide Content

CR supplementation did not affect protein or ash content (*p* > 0.05), but it significantly increased moisture content (*p* < 0.01) and reduced fat content (*p* < 0.01) compared to the control group (Table 5). While the levels of flavor-related ribonucleotides (IMP, GMP, inosine, hypoxanthine) were not affected by dietary CR (*p* > 0.05).

## 4. Discussion

In this study, broilers receiving different levels of CR had a lower ADFI than the control group, suggesting that CR supplementation may have influenced feed palatability. Despite the reduction in feed intake, cumulative body weight, growth rate, and gain-to-feed ratio remained unaffected, indicating that broilers maintained efficient nutrient utilization. This could be attributed to the bioactive compounds present in *C. sativa*, particularly cannabidiol, tetrahydrocannabinol, and polyphenols, which may enhance digestion and nutrient absorption. Our previous findings [10] demonstrated that CR contains cannabidiol (754 mg/kg), tetrahydrocannabinol (1005 mg/kg), and total phenols (28.29 mM GAE/g), with potent antioxidant properties measured through DPPH (236.94 mM TE/g), ABTS (188.73 mM TE/g), and FRAP (0.04 mM Fe^2^^+^/g) assays.

Concerns regarding the potential toxicity of CR supplementation were addressed in our previous study, which found no significant changes in liver enzyme levels, including alanine aminotransferase, aspartate aminotransferase, and alkaline phosphatase. These results suggest that CR supplementation at the levels tested has no effect on liver function or the general health of broilers [10]. The lack of significant effects on survival and EPI in the present study is further evidence that CR-induced toxicity in broilers is insignificant. Future research should investigate the mechanisms by which CR phytochemicals affect feed palatability and nutrient utilization as well as evaluate the potential long-term effects of supplementation on broiler performance.

Physical characteristics such as pH, meat color, water loss, and tenderness are commonly used indicators of meat quality [22]. Shen et al. [23] reported that higher pH values were associated with the inhibition of glycolysis, resulting in improved tenderness and reduced drip loss. In this study, CR supplementation did not significantly influence the physical properties of breast meat, including pH, color, or water loss. This finding aligns with previous research showing that herbal supplementation often does not alter meat quality parameters [24,25]. For example, Park et al. [25] observed that medicinal plant extracts rich in phenolic compounds and flavonoids did not affect pH, color, drip loss, or tenderness in broiler breast muscle. However, contradictory findings have been reported in studies using cannabidiol-rich *C. sativa* extracts, which increased pH and decreased drip loss, particularly in broilers challenged with *Clostridium perfringens* [7]. These variations may be due to differences in the plant species and the forms in which they were used in the various studies. Regarding color differences, all CR-supplemented groups exhibited ΔE values above 3.5, indicating that the color differences compared to the control were clearly perceptible to the human eye. According to the commonly accepted ΔE interpretation scale, values exceeding 3.5 suggest noticeable color variation, which could potentially influence consumer perception of meat appearance [16].

CR supplementation significantly reduced the concentrations of certain fatty acids, including lauric acid, gondoic acid, and erucic acid in breast meat. However, it did not alter the total concentrations of SFA, MUFA, PUFA, omega-6, or omega-3. These results suggest that while CR supplementation affects certain individual fatty acids, its overall effect on the broader fatty acid profile appears to be limited. Lauric acid, a medium-chain saturated fatty acid, has been associated with pro-inflammatory effects by upregulating inflammation-related genes, including cyclooxygenase-2, and activating the nuclear factor kappa B (NF-κB) signaling pathway [26]. In contrast, some studies suggest that lauric acid may benefit intestinal immune response [27]. In this study, CR supplementation led to a reduction in lauric acid content in broiler meat, which could indicate the combined effects of enhanced antioxidant activity, and a potential anti-inflammatory effect of the bioactive compounds present in CR. Additionally, monounsaturated fatty acids, erucic acid, and gondoic acid have been linked to human cardiovascular risks [28]. The observed reduction in these fatty acids with CR supplementation suggests that dietary phytochemicals may modulate lipid metabolism in broilers, leading to potentially healthier meat composition. However, the exact relationship between phytochemical compounds and fatty acid composition in broiler meat remains unclear. Some studies have found no significant changes in fatty acid profiles when broilers were supplemented with dried herbs such as *Rosmarinus officinalis*, *Origanum vulgare*, or *Hypericum perforatum* [29]. In contrast, encapsulated plant extracts have been reported to decrease SFA content and increase MUFA in broiler meat [30]. These variations may be attributed to differences in the antioxidant properties, dosage, and bioavailability of the phytochemicals used.

In this study, the observed increase in free amino acid levels in broilers supplemented with CR may be attributed to its bioactive compounds, such as phenols, flavonoids, and cannabinoids. These compounds are known for their potent antioxidant properties [31,32], which help reduce oxidative stress and protect amino acids from oxidation and degradation [33]. Additionally, our previous study confirmed that dietary CR improved intestinal morphology, as evidenced by a higher villus height-to-crypt depth ratio [10], which may facilitate nutrient digestion and amino acid absorption. Furthermore, the anti-inflammatory properties of cannabinoids [34,35] could contribute to reducing muscle damage, thereby preserving amino acids for muscle protein synthesis. CR supplementation may also alter gut microbiota composition [36], potentially promoting beneficial microbes that produce bioactive metabolites, which, in turn, may improve nutrient utilization and amino acid metabolism. Importantly, increased levels of Glu and Asp, which are known to enhance umami flavor, suggest that CR supplementation may improve the sensory quality of broiler meat [37]. The observed relationship between CR and flavor-enhancing amino acids could result from antioxidant protection, improved protein metabolism, enhanced gut health, and metabolic regulation by bioactive compounds. These mechanisms, working synergistically, likely contribute to the enrichment of specific amino acids, thereby enhancing broiler meat’s taste and nutritional value.

CR supplementation significantly increased moisture content and reduced fat content compared to the control group, with no effect on protein or ash content. The lower fat content in the CR-supplemented group may be attributed to the lipid-lowering effect of cannabidiol (CBD) compounds derived from *C. sativa*. Previous studies have shown that CBD can lower lipid levels in various models, including hepatocytes, zebrafish larvae, and obese mice, by activating the extracellular-signaling-regulated kinase 1/2 (ERK1/2) pathway and increasing 5′-adenosine monophosphate-activated protein kinase (AMPK) activity [38]. These pathways were associated with reduced lipogenesis and increased lipolysis, suggesting a regulatory role of CBD in lipid metabolism. Additionally, polyphenolic compounds were known to activate AMPK signaling in metabolic organs, leading to the downregulation of key lipogenic genes, including stearoyl-CoA desaturase-1, fatty acid synthase, acetyl-CoA carboxylase, and fatty acid elongase 6 in broiler livers [39,40]. Based on these findings, it is hypothesized that CBD and polyphenolic compounds in CR may contribute to EE content in broiler meat. Additionally, the increased moisture content in CR-fed groups could be attributed to the inverse relationship between meat moisture and fat content, which directly impacts meat juiciness [41]. Similar findings have been reported by Al-Hijazeen et al. [42], who observed higher moisture content and lower fat levels in broiler meat from birds supplemented with *Origanum syriacum*. However, previous studies on *C. sativa* supplementation in poultry had yielded inconsistent results. Konieczka et al. [6] found no significant effects of 3% *C. sativa* extract on broiler breast meat composition, while Bień et al. [7] reported higher fat content and lower moisture and protein levels in CBD-supplemented broilers, especially under stress conditions. Variations could influence these differences in bioactive compound composition, dosage, experimental conditions, and individual metabolic responses.

During slaughter and post-mortem aging, adenosine triphosphate (ATP) degradation leads to the formation of key metabolites such as IMP, GMP, hypoxanthine, and inosine, which influence meat flavor [43]. IMP is a major contributor to umami taste, while GMP is complementary in flavor enhancement. In contrast, inosine and hypoxanthine may impart a bitter taste, potentially affecting meat palatability [43]. Natural antioxidants have been suggested to modulate ribonucleotide content in meat, as observed in a study by Li et al. [44], where Yingshan yunwu tea polysaccharides increased IMP and GMP levels in chicken meat. However, in this study, CR supplementation did not affect ribonucleotide content. Similar findings were reported by Ding et al. [22], who found that dietary *Illicium verum* supplementation did not enhance ribonucleotide concentrations in broiler meat. The differences in outcomes across studies may be attributed to variations in bioactive compound composition, antioxidant properties, and dosages used in different experimental settings.

## 5. Conclusions

This study suggests that 2% CR supplementation significantly improved broiler meat’s fatty acid profile, free amino acid content, and chemical composition without compromising performance or physical properties. In addition, CR supplementation appears to positively affect the free amino acids responsible for the umami flavor in breast meat. Therefore, CR could be an effective feed additive to enhance broiler meat’s flavor and nutritional properties. Further studies are needed to verify these results and to understand the mechanisms involved.

## Figures and Tables

**Table 1:** An analysis of the chemical composition of experimental diets containing different levels of *Cannabis sativa* residues (CR).

Variables	Starter Diets (1–23 Days)				Finisher Diets (24–40 Days)			
	CR				CR			
	0%	0.5%	1%	2%	0%	0.5%	1%	2%
Proximate composition (%)								
Dry matter	91.57	91.45	91.18	91.33	90.90	90.82	90.88	90.91
Crude protein	22.08	21.06	21.63	22.18	21.76	20.70	20.25	21.53
Ether extract	5.31	6.16	5.69	5.31	5.59	4.50	4.74	4.74
Ash	6.22	6.59	6.76	6.86	5.75	5.47	5.46	5.92
Crude fiber	3.53	3.50	3.61	3.46	3.22	3.32	2.99	3.21
Gross energy (Cal/g)	4002	3892	3986	3927	3950	3899	3869	3829
Cannabinoid (mg/Kg)								
THC ^1^	nd ^3^	8.6	18.1	30.7	nd	8	18	37.6
CBD ^2^	nd	1.6	2.4	2.9	nd	1.2	1.3	4.8

^1^ THC: Delta-9-tetrahydrocannabinol. ^2^ CBD: Cannabidiol. ^3^ nd: not detected.

**Table 2:** Physicochemical properties of breast meat in broilers supplemented with *C. sativa* residue ^1^.

Variables	Treatments				SEM	*p*-Value
	Control	0.5% CR	1% CR	2% CR		
pH_3_	6.55	6.58	6.50	6.49	0.02	0.439
pH_24_	6.14	6.24	6.10	6.22	0.02	0.088
Lightness (L*)	52.92	52.96	53.33	53.81	0.34	0.791
Redness (a*)	−1.61	−1.40	−1.23	−1.53	0.07	0.170
Yellowness (b*)	6.66	7.46	7.67	7.70	0.21	0.243
Drip loss (%)	3.03	3.16	3.31	3.15	0.09	0.782
Thawing loss (%)	7.43	7.61	8.42	8.71	0.32	0.418
Cooking loss (%)	10.71	11.04	11.78	10.76	0.37	0.723
Shear force (kG)	2.07	2.02	2.24	1.87	0.08	0.414

^1^ Data represent the meaning values of 16 replicates per treatment.

**Table 3:** Fatty acid composition of breast meat in broilers supplemented with *C. sativa* residue ^1^.

Variables	Treatments				SEM	*p*-Value
	Control	0.5% CR	1% CR	2% CR		
C10:0	0.011	0.011	0.013	0.015	0.001	0.721
C12:0	0.612 ^a^	0.512 ^b^	0.523 ^b^	0.563 ^a,b^	0.012	0.005
C14:0	0.947	0.899	0.899	0.926	0.010	0.281
C14:1	0.216	0.164	0.178	0.197	0.010	0.274
C15:0	0.051	0.050	0.047	0.053	0.003	0.929
C16:0	26.614	26.573	26.593	26.885	0.160	0.899
C16:1	7.960	6.904	7.088	6.422	0.308	0.367
C17:0	0.063	0.067	0.059	0.074	0.004	0.514
C17:1	0.039	0.037	0.053	0.050	0.004	0.357
C18:0	5.293	5.753	5.534	5.581	0.088	0.333
C18:1n9c	41.685	41.704	42.576	42.256	0.225	0.434
C18:2n6c	14.893	15.823	14.994	15.498	0.272	0.609
C20:0	0.073	0.068	0.059	0.064	0.004	0.545
C18:3n6	0.151	0.152	0.156	0.179	0.008	0.609
C20:1n9	0.177 ^a^	0.071 ^b^	0.105 ^a,b^	0.063 ^b^	0.014	0.006
C18:3n3	0.659	0.511	0.686	0.587	0.041	0.455
C21:0	0.020	0.019	0.017	0.019	0.001	0.955
C20:2	0.118	0.103	0.050	0.140	0.019	0.376
C22:0	0.014	0.011	0.019	0.022	0.002	0.057
C20:3n6	0.071	0.089	0.084	0.071	0.005	0.550
C20:3n3	0.001	0.002	0.045	0.032	0.010	0.326
C20:4n6	0.055	0.065	0.062	0.063	0.005	0.927
C22:1n9	0.008 ^a,b^	0.010 ^a^	0.003 ^b,c^	0.001 ^c^	0.001	0.001
Others	0.269	0.402	0.157	0.239	0.044	0.118
SFA	33.697	33.962	33.762	34.200	0.192	0.807
MUFA	50.340	49.277	50.143	49.214	0.299	0.436
PUFA	15.846	16.659	16.045	16.447	0.294	0.773
Omega-6	15.185	16.145	15.310	15.825	0.279	0.606
Omega-3	0.660	0.514	0.736	0.622	0.042	0.323

^a,b,c^ Means with different superscript letters within rows differ significantly (*p* < 0.05). ^1^ g/100 g of total fatty acids; data represent mean values of 8 replicates per treatment.

**Table 4:** Free amino acid content in broilers supplemented with *C. sativa* residue ^1^.

Variables	Treatments				SEM	*p*-Value
	Control	0.5% CR	1% CR	2% CR		
Aspartic acid	0.037 ^a,b^	0.043 ^a,b^	0.031 ^b^	0.046 ^a^	0.002	0.011
Glutamic acid	0.279	0.291	0.340	0.370	0.014	0.083
Histidine	0.443	0.373	0.495	0.596	0.036	0.159
Serine	0.101 ^b^	0.135 ^a,b^	0.122 ^a,b^	0.159 ^a^	0.006	0.005
Arginine	1.730	2.005	1.578	1.779	0.060	0.082
Glycine	0.608 ^a,b^	0.696 ^a^	0.585 ^b^	0.578 ^b^	0.017	0.045
Threonine	0.272	0.351	0.358	0.407	0.023	0.244
Alanine	0.168	0.191	0.248	0.257	0.015	0.111
Proline	0.072 ^b^	0.081 ^a,b^	0.087 ^a,b^	0.110 ^a^	0.005	0.017
Lysine	0.426	0.363	0.350	0.372	0.016	0.361
Valine	0.059	0.058	0.061	0.072	0.003	0.193
Methionine	0.050 ^b^	0.062 ^a,b^	0.056 ^b^	0.075 ^a^	0.003	0.002
Isoleucine	0.099	0.098	0.105	0.118	0.004	0.274
Leucine	0.176	0.197	0.193	0.235	0.008	0.063
Phenylalanine	0.109 ^b^	0.127 ^a,b^	0.123 ^a,b^	0.153 ^a^	0.005	0.027
Tryptophane	0.061	0.049	0.012	0.035	0.010	0.347
EAA ^2^	3.424	3.681	3.328	3.841	0.098	0.232
NEAA ^2^	1.265 ^b^	1.438 ^a,b^	1.415 ^a,b^	1.519 ^a^	0.033	0.043
Human taste classification ^3^						
Sweetness	1.220	1.455	1.398	1.513	0.040	0.141
Bitterness	3.040	3.220	2.903	3.326	0.076	0.211
Umami	0.316	0.334	0.371	0.416	0.015	0.083

^a,b^ Means with different superscript letters within rows differ significantly (*p* < 0.05). ^1^ g/100 g of breast meat; data represent mean values of 8 replicate per treatment. ^2^ EAA (essential amino acid) = arginine + histidine + isoleucine + leucine + lysine + methionine + phenylalanine + threonine + valine + tryptophan, NEAA (non-essential amino acid) = alanine + aspartic acid + glutamic acid + glycine + serine + proline. ^3^ Sweet = glycine + alanine + proline + serine + threonine; Bitter = histidine + arginine + isoleucine + leucine + lysine + phenylalanine + valine; Umami = glutamic acid + aspartic acid.

**Table 5:** Chemical composition and ribonucleotide content of breast meat in broilers supplemented with *C. sativa* residue ^1^.

Variables	Treatments				SEM	*p*-Value
	Control	0.5%CR	1%CR	2%CR		
Chemical composition (%)						
Moisture	73.78 ^b^	74.58 ^a^	74.32 ^a,b^	74.79 ^a^	0.10	0.003
Protein	24.66	23.67	24.24	23.87	0.14	0.057
Ash	1.33	1.32	1.30	1.29	0.02	0.380
Ether extract	0.73 ^a^	0.56 ^a,b^	0.70 ^a,b^	0.52 ^b^	0.03	0.007
Ribonucleotide content (mg/100 g)						
Guanosine monophosphate	5.01	5.56	5.79	4.64	0.26	0.410
Inosine monophosphate	299.46	269.43	251.87	240.44	19.91	0.792
Hypoxanthine	27.33	35.11	28.80	24.86	1.76	0.203
Inosine	119.51	103.46	107.42	124.30	7.10	0.734

^a,b^ Means with different superscript letters within rows differ significantly (*p* < 0.05). ^1^ Data represent mean values of 8 replicates per treatment (chemical composition) and 6 replicates per treatment (ribonucleotide content).

## Data Availability

The data that support the findings of this study are available upon reasonable request.
